# Supplementary material for: Concurrent use of low complexity automated NAATs for TB diagnosis and detection of resistance: A cost-effectiveness analysis
Source: PLOS Glob Public Health. 2025 Aug 5;5(8):e0004930. doi: 10.1371/journal.pgph.0004930 (PMC12324103; doi:10.1371/journal.pgph.0004930)
Supplement: S1 Table — (DOCX) [file pgph.0004930.s001.docx]

**S1 Table. Epidemiological model parameters**

| **Target Group** | **Parameter** | **Malawi** | **Philippines** | **Reference** |
| --- | --- | --- | --- | --- |
|  |  | **Base data** | **Base data** |  |
| **Children** | Prevalence of active TB disease among presumptive TB children | 0.136  (0.073 -0.450) | 0.154  (0.080 -0.450) | [1–4] |
|  | Prevalence of RRTB among active TB children | 0.109  (0.082 -0.136) | 0.0415  (0.032-0.054) | [1–6] |
|  | Proportion of under-five presumptive children | 0.41  (0.340-0.500) | 0.41  (0.340-0.500) | [7] |
|  | Probability of providing sputum sample by under-five years presumptive children | 0.024  (0.020-0.027) | 0.024  (0.020-0.027) | [8] |
|  | Probability of providing sputum sample by five years and above presumptive children | 0.377  (0.254-0.512) | 0.377  (0.254-0.512) | [8] |
|  | Probability of death among DS TB children under FLD | 0.019  (0.012-0.029) | 0.019  (0.012-0.029) | [8–11] |
|  | Probability of death among RR TB children under SLD | 0.08  (0.040-0.110) | 0.06  (0.040-0.110) | [12–14] |
|  | Probability of death among DS and DR TB children under 5 years not under ATT | 0.436  (0.368 -0.506) | 0.436  (0.368 -0.506) | [8–11] |
|  | Probability of death among DS and DR TB children five years and above not under ATT | 0.149  (0.115-0.191) | 0.149  (0.115-0.191) | [8–11] |
| **PLHIV** | Prevalence of active TB disease among presumptive TB PLHIV | 0.24  (0.150-0.450) | 0.24  (0.150-0.450) | [15–19] |
|  | Prevalence of RRTB among active TB PLHIV | 0.023  (0.010-0.050) | 0.025  (0.020 -0.054) | [5,6,18,19] |
|  | Probability of providing respiratory samples PLHIV | 0.5  (0.300-0.900) | 0.5  (0.300-0.900) | [18–20] |
|  | Probability of death among DS TB PLHIV under FLD | 0.13  (0.064-0.200) | 0.1  (0.080-0.140) | [21,22] |
|  | Probability of death among RR TB PLHIV under SLD | 0.21  (0.130-0.420) | 0.21  (0.130-0.420) | [23] |
|  | Probability of death among DS and DR TB PLHIV not under ATT | 0.9  (0.750-1.000) | 0.9  (0.750-1.000) | [22,24] |
| **CLHIV** | Prevalence of active TB disease among presumptive TB CLHIV | 0.24  (0.15-0.45) | 0.24  (0.15-0.45) | [15–19] |
|  | Prevalence of RRTB among active TB CLHIV | 0.11  (0.08-0.14) | 0.0415  (0.032-0.054) | [1–6] |
|  | Proportion of under-five presumptive children | 0.41  (0.34-0.50) | 0.41  (0.34-0.50) | [7] |
|  | Probability of providing sputum sample by under-five years presumptive children | 0.024  (0.020-.027) | 0.024  (0.020-.027) | [8] |
|  | Probability of providing sputum sample by five years and above presumptive children | 0.38  (0.250-0.51) | 0.38  (0.250-0.51) | [8] |
|  | Probability of death among DS TB CLHIV under FLD | 0.019  (0.012-0.03) | 0.019  (0.012-0.03) | [8–11] |
|  | Probability of death among RR TB CLHIV under SLD | 0.08 (0.040-0.11) | 0.08 (0.040-0.11) | [12–14] |
|  | Probability of death among DS and DR TB CLHIV not under ATT | 0.9  (0.75-1) | 0.9  (0.75-1) | [22,24] |
|  |  |  |  |  |

TB: Tuberculosis; RR: Rifampicin Resistant; DS: Drugs Susceptible; DR: Drug Resistant; FLD: Firstline Drugs; SLD: Second line Drugs; ATT: Anti-TB Treatment; PLHIV: People Living with HIV; CLHIV: Children Living with HIV; RR: Rif. Resistant
