# Supplementary material for: Concurrent use of low complexity automated NAATs for TB diagnosis and detection of resistance: A cost-effectiveness analysis
Source: PLOS Glob Public Health. 2025 Aug 5;5(8):e0004930. doi: 10.1371/journal.pgph.0004930 (PMC12324103; doi:10.1371/journal.pgph.0004930)
Supplement: S2 Table — (DOCX) [file pgph.0004930.s002.docx]

**S2 Table. Diagnostic accuracy model parameters**

| **Target Group** | **Parameter** | **Point Estimate** | **Reference** |
| --- | --- | --- | --- |
| **Children** | Sensitivity of LC-aNAAT concurrent testing (respiratory and stool) | 0.799  (0.678-0.898) | [25] |
|  | Specificity of LC-aNAAT concurrent testing (respiratory and stool) | 0.934  (0.872-0.97) | [25] |
|  | Sensitivity of LC_aNAAT in respiratory sample | 0.726  (0.59-0.846) | [25] |
|  | Specificity of LC-aNAAT in respiratory sample | 0.95  (0.906-0.977) | [25] |
|  | Sensitivity of LC-aNAAT in stool | 0.563  (0.429-0.699) | [25] |
|  | Specificity of LC-aNAAT in stool | 0.975  (0.948-0.99) | [25] |
|  | Sensitivity of clinical diagnosis | 0.63  (0.59-0.66) | [26] |
|  | Specificity of clinical diagnosis | 0.90  (0.88-0.92) | [26] |
| **PLHIV** | Sensitivity of LC_aNAAT concurrent testing with LF-LAM | 0.775  (0.73-0.81) | [25] |
|  | Specificity of LC_aNAAT concurrent testing with LF-LAM | 0.894  (0.86-0.92) | [25] |
|  | Sensitivity of LC_aNAAT in respiratory sample | 0.68  (0.61-0.75) | [25] |
|  | Specificity of LC-aNAAT in respiratory sample | 0.967  (0.96-0.98) | [25] |
|  | Sensitivity of LF-LAM among PLHIV | 0.391  (0.33-0.46) | [25] |
|  | Specificity of LF-LAM among PLHIV | 0.919  (0.89-0.94) | [25] |
|  | Sensitivity of clinical diagnosis | 0.61  (0.55-0.67) | [21,27] |
|  | Specificity of clinical diagnosis | 0.69  (0.66-0.73) | [21,27] |
| **CLHIV** | Sensitivity of LC_aNAAT concurrent testing with respiratory and stool samples and LF LAM in urine among CLHIV | 0.776  (0.60-.89) | [25] |
|  | Specificity of LC_aNAAT concurrent testing with respiratory and stool samples and LF LAM in urine among CLHIV | 0.839  (0.74-0.90) | [25] |
|  | Sensitivity of LC-aNAAT in respiratory sample among CLHIV | 0.68  (0.54-0.80) | [25] |
|  | Specificity of LC-aNAAT in respiratory sample among CLHIV | 0.939  (0.90-0.96) | [25] |
|  | Sensitivity of clinical diagnosis of presumptive TB CLHIV with LC-aNAAT negative | 0.61  (0.58-0.64) | [28,29] |
|  | Specificity of clinical diagnosis of presumptive TB CLHIV with LC-aNAAT negative | 0.89  (0.87-0.90) | [28,29] |
|  | Sensitivity of LC-aNAAT for stool sample CLHIV | 0.687  (0.51-0.82) | [25] |
|  | Specificity of LC-aNAAT for stool sample among CLHIV | 0.995  (0.83-1.00) | [25] |
|  | Sensitivity of LF-LAM in urine among CLHIV to detect M. TB | 0.274  (0.13-0.47) | [25] |
|  | Specificity of LF-LAM in urine among CLHIV to detect M. TB | 0.882  (0.78-0.94) | [25] |

LC_aNAAT: Low complexity automated nucleic acid amplification tests; PLHIV: People Living with HIV; CLHIV: Children Living with HIV; M. TB: Mycobacterium Tuberculosis; LF-LAM: Lateral Flow Lipoarabinomannan
