# Supplementary material for: Concurrent use of low complexity automated NAATs for TB diagnosis and detection of resistance: A cost-effectiveness analysis
Source: PLOS Glob Public Health. 2025 Aug 5;5(8):e0004930. doi: 10.1371/journal.pgph.0004930 (PMC12324103; doi:10.1371/journal.pgph.0004930)
Supplement: S3 Table — (DOCX) [file pgph.0004930.s003.docx]

**S3 Table. DALYs model parameter**

| **Parameter** | **Point Estimate** | **Reference** |
| --- | --- | --- |
| Children with TB (DS or RR), treatment failure, LTFU, or without treatment, and surviving | 0.66  (0.528–0.792) | [36,37] |
| Children with DSTB or RRTB, treatment success and surviving | 0.33  (0.224–0.454) |  |
| CLHIV receiving ART without TB | 0.078  (0.057–0.123) |  |
| CLHIV with DSTB or RRTB, treatment success and surviving | 0.408  (0.274–0.549) |  |
| Children with TB (DS or RR), treatment failure, LTFU, or without treatment, and surviving | 0.66  (0.528–0.792) |  |
| PLHIV receiving ART without TB | 0.078  (0.057–0.123) |  |
| PLHIV with DSTB or RRTB, treatment success and surviving | 0.408  (0.274–0.549) |  |
| PLHIV with TB (DS or RR), treatment failure, LTFU, or without treatment, and surviving | 0.66  (0.528–0.792) |  |

ART: Antiretroviral therapy; CLHIV: Children living with HIV; DSTB: Drug-susceptible tuberculosis; LTFU: Lost to follow-up; PLHIV: People living with HIV; RR: Rifampicin-resistant; RRTB: Rifampicin-resistant tuberculosis; TB: Tuberculosis; DS: Drug-susceptible.
