# Supplementary material for: Concurrent use of low complexity automated NAATs for TB diagnosis and detection of resistance: A cost-effectiveness analysis
Source: PLOS Glob Public Health. 2025 Aug 5;5(8):e0004930. doi: 10.1371/journal.pgph.0004930 (PMC12324103; doi:10.1371/journal.pgph.0004930)
Supplement: S4 Table — (DOCX) [file pgph.0004930.s004.docx]

**S4 Table. Cost model parameters (US$)**

| **Target Group** | **Parameter** | **Malawi** | **Philippines** | **Reference** |
| --- | --- | --- | --- | --- |
|  |  | **Base data** | **Base data** |  |
| Children and CLHIV | Per unit test cost of LC_aNAAT on respirator sample | 33  (20-35) | 25  (21-29) | [18–20,30,31] |
|  | Per unit test cost of LF-LAM | 6  (3-20) | 8  (3-20) | [18,19,31] |
|  | Cost of FLD | 541  (367-715) | 392  (294-490) | [8,10,20,31,32] |
|  | Cost of SSTR | 1928  (942-2915) | 1329  (665-1994) | [31,33,34] |
|  | Cost of ART | 167  (83-292) | 140  (70-210) | [18,19,35] |
| PLHIV | Per unit test cost of LC_aNAAT on respirator sample | 33  (20-35) | 25  (21-29) | [18–20,30,31] |
|  | Per unit test cost of LF-LAM | 6  (3-20) | 8  (3-20) | [18,19,31] |
|  | Cost of FLD | 193  (154-236) | 148  (108-185) | [8,10,20,31,32] |
|  | Cost of BPALM | 3520  (1760-5280) | 1364  (682-2046) | [31,33,34] |
|  | Cost of ART | 167  (83-292) | 140  (70-210) | [18,19,35] |

LC_aNAAT: Low complexity automated nucleic acid amplification tests; PLHIV: People Living with HIV; CLHIV: Children Living with HIV; LF-LAM: Lateral Flow Lipoarabinomannan; FLD: First-Line Drugs; SSTR: Standardized Short-Term regimen; ART: Antiretroviral treatment; BPALM: Bedaquiline, Pretomanid, Linezolid, and Moxifloxacin
