# Supplementary material for: Concurrent use of low complexity automated NAATs for TB diagnosis and detection of resistance: A cost-effectiveness analysis
Source: PLOS Glob Public Health. 2025 Aug 5;5(8):e0004930. doi: 10.1371/journal.pgph.0004930 (PMC12324103; doi:10.1371/journal.pgph.0004930)
Supplement: S5 Table — (DOCX) [file pgph.0004930.s005.docx]

**S5 Table. Additional TB Cases Diagnosed and Deaths Averted per 1,000 Individuals by Target Group and Country**

| Target group | Country | Additional TB Cases Diagnosed per 1,000 individuals | Additional deaths Averted per 1,000 individuals |
| --- | --- | --- | --- |
| Children | Malawi | 40 | 6 |
|  | Philippines | 43 | 7 |
| PLHIV | Malawi | 63 | 18 |
|  | Philippines | 63 | 19 |
| CLHIV | Malawi | 136 | 58 |
|  | Philippines | 137 | 53 |

TB: Tuberculosis; PLHIV: People Living with HIV; CLHIV: Children Living with HIV
