# Supplementary material for: Concurrent use of low complexity automated NAATs for TB diagnosis and detection of resistance: A cost-effectiveness analysis
Source: PLOS Glob Public Health. 2025 Aug 5;5(8):e0004930. doi: 10.1371/journal.pgph.0004930 (PMC12324103; doi:10.1371/journal.pgph.0004930)
Supplement: S1 Fig — (DOCX) [file pgph.0004930.s006.docx]

**
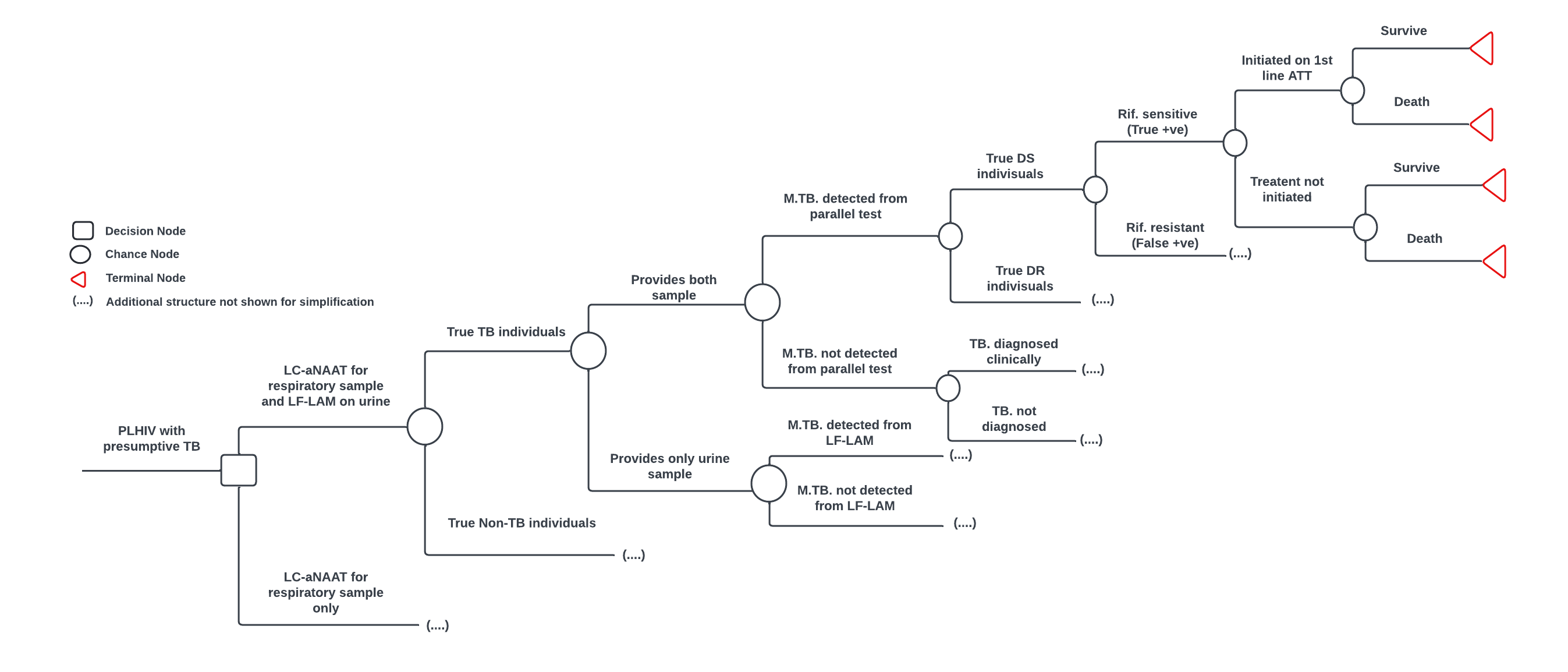
**

**S1 Fig. Simplified model decision structure on the concurrent use of LC-aNAAT among PLHIV:**  Two strategies will be compared: Testing for TB with LC-aNAAT in respiratory sample and LF-LAM on urine vs. single use of LC-aNAAT in respiratory sample among adult PLHIV with presumptive TB. Schematically these strategies are separated by a square representing decision node. The circle represents chance nodes where individuals may experience one of several possible events shown on subsequent lines. Dotted lines represent model structure omitted for simplicity. The triangle symbol represents terminal node. may experience one of several possible events shown on subsequent lines. TB: Tuberculosis; LC-aNAAT: Low complexity automated nucleic acid amplification tests; DS: Drugs susceptible; DR: Drugs resistant; Rif: Rifampicin; RS: Rif. Sensitive; RR: Rif. Resistant
