# Supplementary material for: Concurrent use of low complexity automated NAATs for TB diagnosis and detection of resistance: A cost-effectiveness analysis
Source: PLOS Glob Public Health. 2025 Aug 5;5(8):e0004930. doi: 10.1371/journal.pgph.0004930 (PMC12324103; doi:10.1371/journal.pgph.0004930)
Supplement: S5 Fig — (DOCX) [file pgph.0004930.s010.docx]

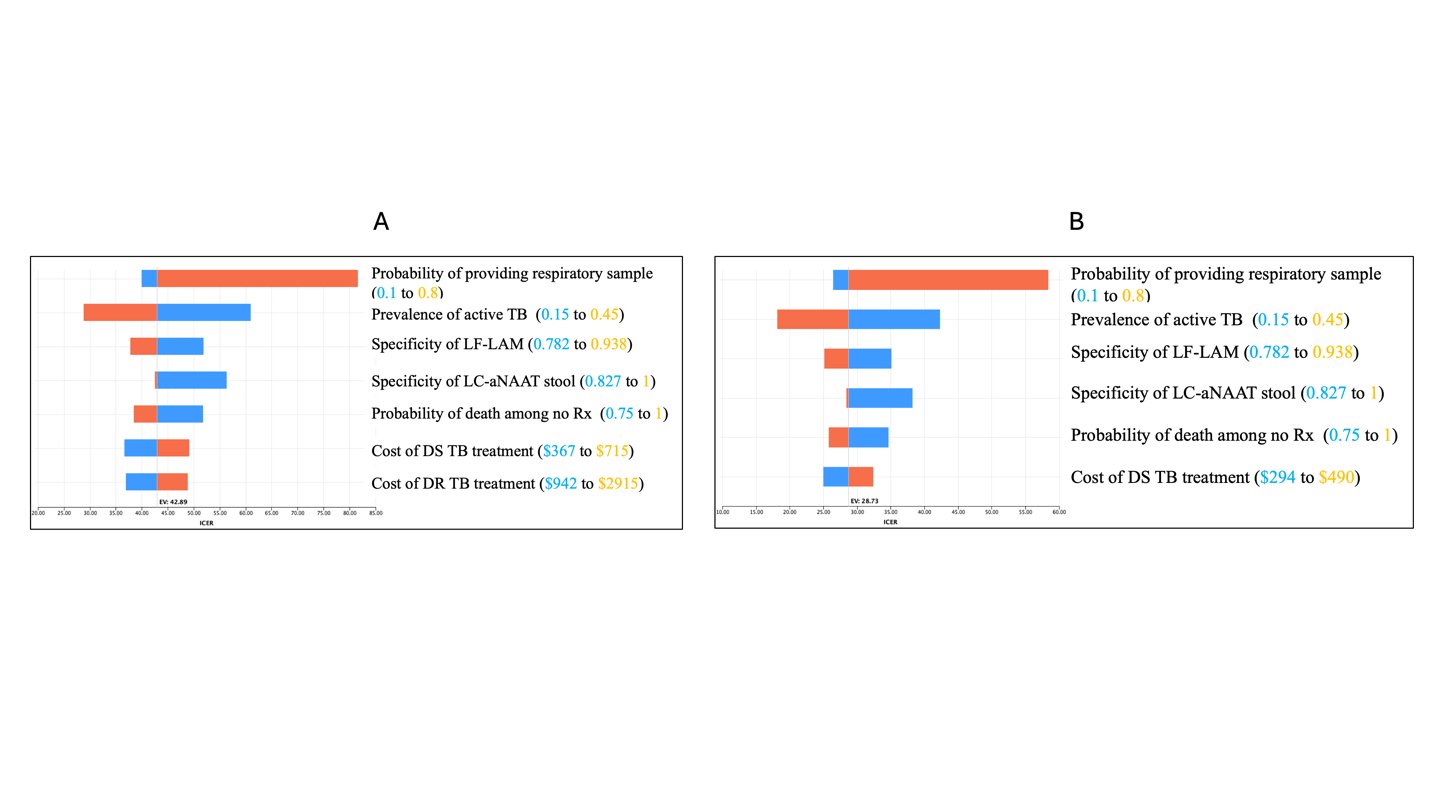


**S5 Fig. One-way sensitivity analysis on the concurrent use of LC-aNAAT among CLHIV in Malawi (Panel A) and the Philippines (Panel B):** All parameters were varied and only those parameters that resulted in a change of +/-10% in the incremental cost-effectiveness ratio are shown. Blue bars correspond to the incremental cost-effectiveness of LC-aNAAT in concurrent testing among CLHIV in Malawi and the Philippines relative to respiratory sample testing, at the low value of the specified parameter range. Red bars correspond to the incremental cost-effectiveness at the high value of that range, holding all other parameter values constant. TB: Tuberculosis; LC-aNAAT: Low complexity automated nucleic acid amplification tests; CLHIV: Children Living with HIV
