# Supplementary material for: Concurrent use of low complexity automated NAATs for TB diagnosis and detection of resistance: A cost-effectiveness analysis
Source: PLOS Glob Public Health. 2025 Aug 5;5(8):e0004930. doi: 10.1371/journal.pgph.0004930 (PMC12324103; doi:10.1371/journal.pgph.0004930)
Supplement: S6 Fig — (DOCX) [file pgph.0004930.s011.docx]

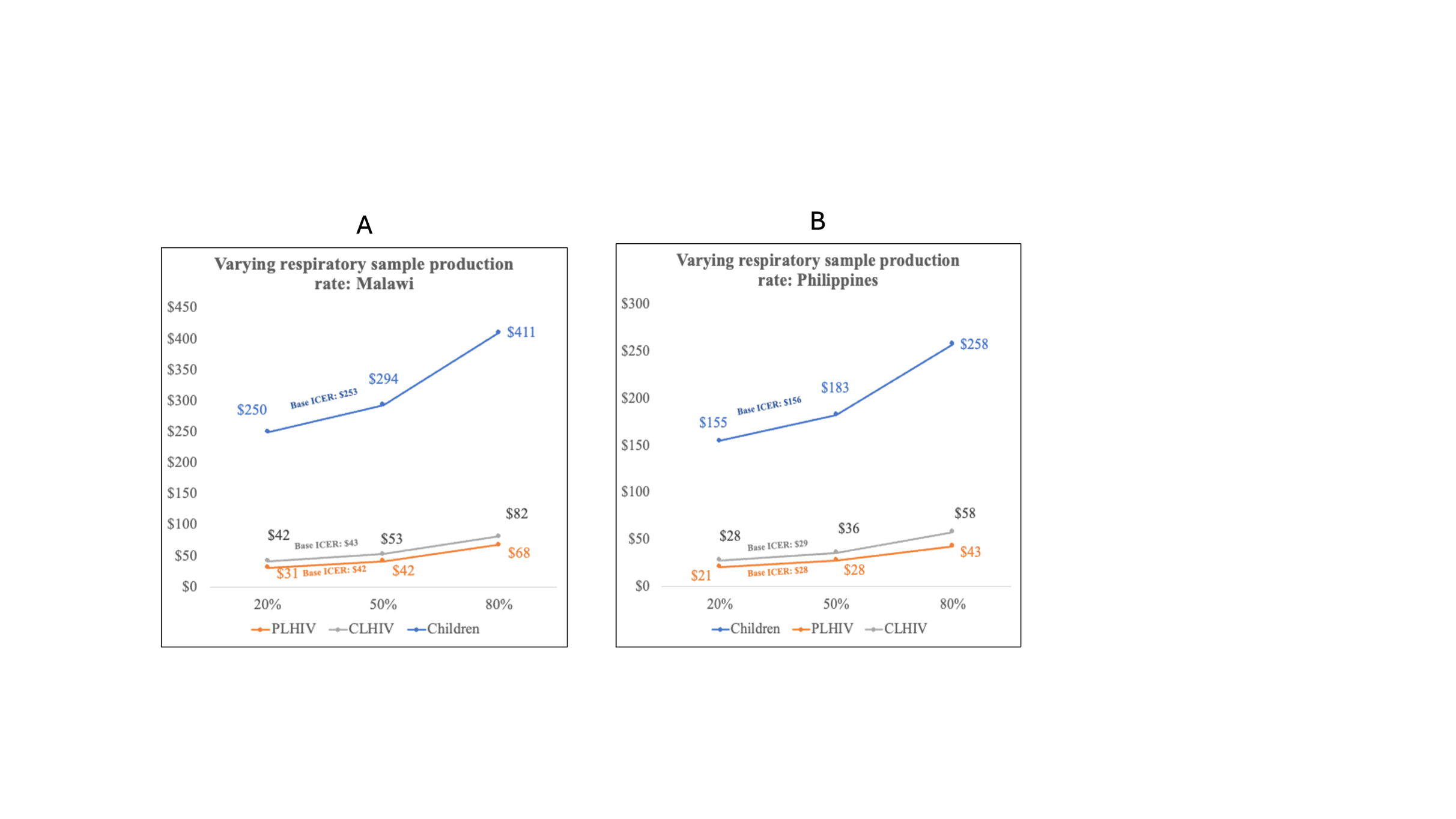


**S6 Fig. Scenario Analysis – Varying respiratory sample production rate Malawi (Panel A) and the Philippines (Panel B):** This figure shows how the ICER changes with varying proportions of respiratory sample production rates (20%, 50%, and 80%) among children, PLHIV, and CLHIV in Malawi and the Philippines. ICER: Incremental Cost-Effectiveness Ratio; PLHIV: People Living with HIV; CLHIV: Children Living with HIV
