# Supplementary material for: Concurrent use of low complexity automated NAATs for TB diagnosis and detection of resistance: A cost-effectiveness analysis
Source: PLOS Glob Public Health. 2025 Aug 5;5(8):e0004930. doi: 10.1371/journal.pgph.0004930 (PMC12324103; doi:10.1371/journal.pgph.0004930)
Supplement: S1 Text — (DOCX) [file pgph.0004930.s013.docx]

**Reference**

1. Arega B, Menbere F, Getachew Y. Prevalence of rifampicin resistant Mycobacterium tuberculosis among presumptive tuberculosis patients in selected governmental hospitals in Addis Ababa, Ethiopia. BMC Infect Dis. 2019;19. doi:10.1186/s12879-019-3943-1

2. Dejene TA, Hailu GG, Kahsay AG, Wasihun AG. Pulmonary Tuberculosis and Rifampicin Resistant Mycobacterium Tuberculosis in Children and Adolescents using Gene Xpert MTB/RIF Assay in Tigray, Northern Ethiopia. Infect Drug Resist. 2023;16. doi:10.2147/IDR.S433789

3. Kizito S, Nakalega R, Nampijja D, Atuheire C, Amanya G, Kibuuka E, et al. High burden of pulmonary tuberculosis and missed opportunity to initiate treatment among children in Kampala, Uganda. Afr Health Sci. 2022;22. doi:10.4314/ahs.v22i4.66

4. Aldaba JG, Ama MCG, Salonga AM, Sylim PG, Sarol JN, Lopez AL. Diagnosis and management of childhood tuberculosis in public health clinics in a rural area in the Philippines: Results from a community surveillance. Acta Med Philipp. 2018;52. doi:10.47895/amp.v52i4.355

5. WHO. TB country, regional and global profile .

6. SECOND NATIONAL DRUG RESISTANCE SURVEY ON TUBERCULOSIS IN THE PHILIPPINES 2 SECOND NATIONAL DRUG RESISTANCE SURVEY ON TUBERCULOSIS IN THE PHILIPPINES.

7. Yerramsetti S, Cohen T, Atun R, Menzies NA. Global estimates of paediatric tuberculosis incidence in 2013–19: a mathematical modelling analysis. Lancet Glob Health. 2022;10. doi:10.1016/S2214-109X(21)00462-9

8. Mafirakureva N, Klinkenberg E, Spruijt I, Levy J, Shaweno D, De Haas P, et al. Xpert Ultra stool testing to diagnose tuberculosis in children in Ethiopia and Indonesia: a model-based cost-effectiveness analysis. BMJ Open. 2022;12. doi:10.1136/bmjopen-2021-058388

9. Snow K, Yadav R, Denholm J, Sawyer S, Graham S. Tuberculosis among children, adolescents and young adults in the Philippines: a surveillance report. Western Pac Surveill Response J. 2018;9. doi:10.5365/wpsar.2017.8.4.011

10. Jo Y, Gomes I, Flack J, Salazar-Austin N, Churchyard G, Chaisson RE, et al. Cost-effectiveness of scaling up short course preventive therapy for tuberculosis among children across 12 countries. EClinicalMedicine. 2021;31. doi:10.1016/j.eclinm.2020.100707

11. Jenkins HE, Yuen CM, Rodriguez CA, Nathavitharana RR, McLaughlin MM, Donald P, et al. Mortality in children diagnosed with tuberculosis: a systematic review and meta-analysis. Lancet Infect Dis. 2017;17: 285–295. doi:10.1016/S1473-3099(16)30474-1

12. Chiang C-Y. Review of Programmatic Management of Drug-Resistant Tuberculosis, Philippines MISSION REPORT. 2022.

13. Toft AL, Dahl VN, Sifna A, Ige OM, Schwoebel V, Souleymane MB, et al. Treatment outcomes for multidrug- and rifampicin-resistant tuberculosis in Central and West Africa: a systematic review and meta-analysis. International Journal of Infectious Diseases. 2022. doi:10.1016/j.ijid.2022.08.015

14. Tola HH, Khadoura KJ, Jimma W, Nedjat S, Majdzadeh R. Multidrug resistant tuberculosis treatment outcome in children in developing and developed countries: A systematic review and meta-analysis. International Journal of Infectious Diseases. 2020. doi:10.1016/j.ijid.2020.03.064

15. Sun D, Dorman S, Shah M, Manabe YC, Moodley VM, Nicol MP, et al. Cost utility of lateral-flow urine lipoarabinomannan for tuberculosis diagnosis in HIV-infected African adults. International Journal of Tuberculosis and Lung Disease. 2013;17. doi:10.5588/ijtld.12.0627

16. Zwerling AA, Sahu M, Ngwira LG, Khundi M, Harawa T, Corbett EL, et al. Screening for tuberculosis among adults newly diagnosed with HIV in Sub-Saharan Africa: A cost-effectiveness analysis. Journal of Acquired Immune Deficiency Syndromes. 2015. doi:10.1097/QAI.0000000000000712

17. Orlando S, Triulzi I, Ciccacci F, Palla I, Palombi L, Marazzi MC, et al. Delayed diagnosis and treatment of tuberculosis in HIV+ patients in Mozambique: A cost-effectiveness analysis of screening protocols based on four symptom screening, smear microscopy, urine LAM test and Xpert MTB/RIF. PLoS One. 2018;13. doi:10.1371/journal.pone.0200523

18. Reddy KP, Denkinger CM, Broger T, McCann NC, Gupta-Wright A, Kerkhoff AD, et al. Cost-effectiveness of a Novel Lipoarabinomannan Test for Tuberculosis in Patients with Human Immunodeficiency Virus. Clinical Infectious Diseases. 2021;73. doi:10.1093/cid/ciaa1698

19. Reddy KP, Gupta-Wright A, Fielding KL, Costantini S, Zheng A, Corbett EL, et al. Cost-effectiveness of urine-based tuberculosis screening in hospitalised patients with HIV in Africa: a microsimulation modelling study. Lancet Glob Health. 2019;7. doi:10.1016/S2214-109X(18)30436-4

20. Brümmer LE, Thompson RR, Malhotra A, Shrestha S, Kendall EA, Andrews JR, et al. Cost-effectiveness of Low-complexity Screening Tests in Community-based Case-finding for Tuberculosis. Clinical Infectious Diseases. 2024;78. doi:10.1093/cid/ciad501

21. Fekadu G, Wang Y, You JHS. Standard diagnostics with and without urine-based lipoarabinomannan testing for tuberculosis disease in HIV-infected patients in a high-burden setting–A cost-effectiveness analysis. PLoS One. 2023;18. doi:10.1371/journal.pone.0288605

22. Keim-Malpass J, Heysell SK, Thomas TA, Lobo JM, Mpagama SG, Muzoora C, et al. Decision Analytic Modeling for Global Clinical Trial Planning: A Case for HIV-Positive Patients at High Risk for Mycobacterium tuberculosis Sepsis in Uganda. Int J Environ Res Public Health. 2023;20. doi:10.3390/ijerph20065041

23. Wu Y, Zhang Y, Wang Y, Wei J, Wang W, Duan W, et al. Bedaquiline and Linezolid improve anti-TB treatment outcome in drug-resistant TB patients with HIV: A systematic review and meta-analysis. Pharmacological Research. 2022. doi:10.1016/j.phrs.2022.106336

24. Shah M, Dowdy D, Joloba M, Ssengooba W, Manabe YC, Ellner J, et al. Cost-effectiveness of novel algorithms for rapid diagnosis of tuberculosis in HIV-infected individuals in Uganda. AIDS. 2013;27. doi:10.1097/QAD.0000000000000008

25. Who. Module 3: Diagnosis WHO consolidated guidelines on tuberculosis.

26. Marais BJ, Gie RP, Hesseling AC, Schaaf HS, Lombard C, Enarson DA, et al. A refined symptom-based approach to diagnose pulmonary tuberculosis in children. Pediatrics. 2006;118. doi:10.1542/peds.2006-0519

27. Walusimbi S, Bwanga F, De Costa A, Haile M, Joloba M, Hoffner S. Meta-analysis to compare the accuracy of GeneXpert, MODS and the WHO 2007 algorithm for diagnosis of smear-negative pulmonary tuberculosis. BMC Infect Dis. 2013;13. doi:10.1186/1471-2334-13-507

28. Vonasek BJ, Rabie H, Hesseling AC, Garcia-Prats AJ. Tuberculosis in Children Living With HIV: Ongoing Progress and Challenges. J Pediatric Infect Dis Soc. 2022;11. doi:10.1093/jpids/piac060

29. Vonasek B, Kay A, Devezin T, Bacha JM, Kazembe P, Dhillon D, et al. Tuberculosis symptom screening for children and adolescents living with HIV in six high HIV/TB burden countries in Africa. AIDS. 2021;35. doi:10.1097/QAD.0000000000002715

30. Sohn H, Puri L, Nguyen NAT, Van’t Hoog AH, Nguyen VAT, Nliwasa M, et al. Cost and affordability analysis of TB-LAMP and Xpert MTB/RIF assays as routine diagnostic tests in peripheral laboratories in Malawi and Vietnam. Journal of Global Health Science. 2019;1. doi:10.35500/jghs.2019.1.e22

31. Value TB Data verse. Available: https://dataverse.harvard.edu/dataverse/Value-TB.

32. Capeding TPJ, Rosa JD, Lam H, Gaviola DG, Garfin AMC, Hontiveros C, et al. Cost of TB prevention and treatment in the Philippines in 2017. International Journal of Tuberculosis and Lung Disease. 2022;26. doi:10.5588/ijtld.21.0622

33. Rosu L, Madan JJ, Tomeny EM, Muniyandi M, Nidoi J, Girma M, et al. Economic evaluation of shortened, bedaquiline-containing treatment regimens for rifampicin-resistant tuberculosis (STREAM stage 2): a within-trial analysis of a randomised controlled trial. Lancet Glob Health. 2023;11. doi:10.1016/S2214-109X(22)00498-3

34. Sweeney S, Berry C, Kazounis E, Motta I, Vassall A, Dodd M, et al. Cost-effectiveness of short, oral treatment regimens for rifampicin resistant tuberculosis. PLOS Global Public Health. 2022;2. doi:10.1371/journal.pgph.0001337

35. Shin H, Jo Y, Chaisson RE, Turner K, Churchyard G, Dowdy DW. Cost-effectiveness of a 12 country-intervention to scale up short course TB preventive therapy among people living with HIV. J Int AIDS Soc. 2020;23. doi:10.1002/jia2.25629

36. Global Burden of Disease Collaborative Network. Global Burden of Disease Study 2019 (GBD 2019) Disability Weights. Institute for Health Metrics and Evaluation, Seattle, USA2020 http://ghdx.healthdata.org/record/ihme-data/gbd-2019-disability-weights. In: 2020.

37. Tomeny EM, Hampton T, Tran PB, Rosu L, Phiri MD, Haigh KA, et al. Rethinking Tuberculosis Morbidity Quantification: A Systematic Review and Critical Appraisal of TB Disability Weights in Cost-Effectiveness Analyses. PharmacoEconomics. Adis; 2024. doi:10.1007/s40273-024-01410-x
