# Supplementary material for: Concurrent use of low complexity automated NAATs for TB diagnosis and detection of resistance: A cost-effectiveness analysis
Source: PLOS Glob Public Health. 2025 Aug 5;5(8):e0004930. doi: 10.1371/journal.pgph.0004930 (PMC12324103; doi:10.1371/journal.pgph.0004930)
Supplement: Attachment — Submitted filename: Response to reviewers May 25.docx [file pgph.0004930.s015.docx]

Dear Dr. Devine,

Academic Editor
PLOS Global Public Health

Thank you for your detailed feedback and for the opportunity to revise our manuscript titled " Concurrent use of low complexity automated NAATs for TB diagnosis and detection of resistance: a cost-effectiveness analysis”. We appreciate the constructive comments from the editorial team and reviewers, which have significantly improved the clarity and quality of our work.

Below, we address the journal’s requirements and the reviewers’ comments. All changes have been made in the revised manuscript and are highlighted accordingly.

**Editorial Requirements- Responses**

**1. Competing Interest Statement:**
We have reviewed the amended Competing Interest Statement provided by the journal and confirm that it is accurate.

**2. Data Availability:**
We confirm that all data used in this modeling analysis are derived from publicly available sources. In compliance with PLOS data policies, we have now included all data and parameter values used in the modeling analysis in the **Supplementary Information** files. These include epidemiological estimates, cost data, DALY weights, and all key assumptions. As the study did not involve human participants or primary data collection, there are no ethical or legal restrictions to sharing these data.

**3. Ethics Statement:**
An **Ethics Statement** has been added under the “Study Design” subsection of the Methods section. As this study relied entirely on secondary data from public sources, ethical approval was not required.

**4. Figure Uploads:**
Figures 1 through 7, as referenced in the manuscript, have now been uploaded as **separate, high-resolution files**, and all references to these figures in the text have been verified and retained.

**Reviewer comments and response**

We have provided a detailed, point-by-point response to all reviewer comments. For each comment, we describe the change made and indicate where it has been implemented in the revised manuscript or supplementary materials. Where applicable, figures have been updated, terminology clarified, and grammatical errors corrected.

Reviewer #1: 
Abstract
Consider replacing “measured” with “estimated or evaluated” in ‘”The analysis was conducted from a healthcare system perspective and measured...”

Response: We thank the reviewer for the helpful suggestion. We have replaced the word **“measured”** with **“estimated”** in the sentence.

Methods
I think some brief description of what LC_aNAAT might look like in practice might help to put these findings in context. What tests are assumed to be used? Are these assumed to be universally available throughout all levels of care?

Response: We thank the reviewer for this helpful suggestion. We would like to note that a brief description of LC-aNAAT, including examples of WHO-recommended platforms such as Xpert MTB/RIF Ultra and Truenat, is already included in the Introduction section. To further clarify the assumptions in our model, we have now added the following sentence to the Methods section: *“In this study, LC-aNAAT is assumed to be available at the level of the health system where there is capacity for TB screening and conducting diagnostic tests, consistent with decentralized implementation strategies. However, we did not assume universal availability across all levels of care, recognizing that access to these tools may vary by setting and programmatic capacity.”* This addition helps clarify the practical assumptions regarding availability of LC-aNAAT in the modeled settings.

Results
I think it may be useful to include some more proximal outcomes in the results e.g cases detected and deaths. Although these are not the primary outcomes, I think they are important especially in programmatic settings. Equally important is including some measures of resource use e.g tests done and treatments required.

Response: We appreciate the reviewer’s valuable suggestion to include more proximal outcomes and measures of resource use. In response, we have included additional TB cases diagnosed and deaths averted per 1,000 individuals in Supplemental Table 4. These outcomes provide a clearer understanding of the programmatic impact of the intervention across target populations. While data on resource use such as number of tests and treatments were not the primary focus of this analysis, we agree that they are important for implementation planning and have noted this as an area for future work.

Figure 1: “The diamond symbol represents terminal node” - probably meant to say “triangle”. Figures: Please define the abbreviations in all the figures. Too many needless decimals are provided in the figures and in some cases making it difficult to read the actual values.

Response: We thank the reviewer for these helpful suggestions. We have corrected the figure legend in Figure 1 to state that the triangle symbol represents the terminal node. Additionally, we have updated all figures to define abbreviations and removed unnecessary decimal places to improve clarity and readability.

I think there are too many results presented in the main text and in my opinion it might be useful to focus on the main findings and a few SAs so that the message is not lost in the detail. The rest can be moved to the Appendix.
Response: We appreciate the reviewer’s suggestion. While we have retained all key results in the main text to ensure completeness, we have addressed concerns about presentation and clarity by **merging Tables 1, 2, and 3 into a single table** and by **combining related figures into multi-panel layouts**. These changes were made based on feedback from other reviewers and help to reduce redundancy and improve the flow of results without omitting important findings.

Discussion
The authors state that “these strategies may demand higher short-term expenditures…” - what is the impact of these strategies on the budget? Was a BIA done? I think it’s important to provide some reflection on what resources would be required and whether they would be affordable.

Response: We thank the reviewer for this insightful comment. We acknowledge that while our analysis focused on cost-effectiveness, we did not conduct a formal Budget Impact Analysis (BIA). We have now clarified this in the Discussion section and added a brief reflection on the potential resource requirements and affordability considerations.

Reviewer #2:

On line 376, the table referred to is Supplementary Table 4, not Supplementary Table 3, as well as on line 437. Please include the list of abbreviations in the supplementary tables and figures. Please add the references used in the systematic reviews (SRs?) to Supplementary Table

Response: Thank you for these helpful comments.

- We have corrected the table references on lines 376 and 437 to refer to **Supplementary Table 4** instead of Supplementary Table 3.
- We have also included a **list of abbreviations** in all supplementary tables and figure legends to improve clarity and usability.
- Additionally, we have **added the references used in the systematic reviews** to the relevant Supplementary Table.

Reviewer #3:

The authors evaluated the cost-effectiveness of the concurrent testing that includes low‐complexity automated nucleic acid amplification tests (LC-aNAAT) and antigen‐based lateral flow urine lipoarabinomannan (LF‐LAM) test among three groups of individuals, which were children under 10 years (HIV-negative only? It is not clear), HIV-positive individuals, and HIV-positive children under 10 years, in Malawi and the Philippines.

In the Abstract, it would be good to include that the study was conducted by using the data of Malawi and Philippines, before the results part.

Response: Thank you for these helpful suggestions.

- We have clarified in the **Abstract** that the study was conducted using data from **Malawi and the Philippines** by adding a sentence stating that the analysis used country-specific parameters from published literature and a systematic review conducted for the WHO Guideline Development Group (GDG).
- We also clarified in the **Methods** that the group referred to as **“children under 10 years” includes only HIV-negative children**.

Also, in line 53, it would be better to provide the unabbreviated version of UR – the more common way to address this term is “uncertainty interval (UI)” for clarity.

Response: Thank you for the helpful suggestion. We have replaced “UR” with the more commonly used term **“uncertainty interval (UI)”** throughout the manuscript to ensure clarity and consistency.

The authors used P/CLHIV, PLHIV and CLHIV in different parts of the manuscript interchangeably. For instance, in line 41, it was referred as “… in children and people living with HIV (P/CLHIV)” and in line 109, the same definition was referred as “… among children and people living with HIV (PLHIV)”. For consistency, it would be useful to include the same abbreviation when indicating the same group of individuals throughout the manuscript.

Response: Thank you for pointing out this inconsistency. We have reviewed the manuscript and now use consistent terminology and abbreviations throughout. Specifically, we use **PLHIV** to refer to **adult people living with HIV**, and **CLHIV** to refer to **children living with HIV**. The abbreviation **P/CLHIV** has been removed to avoid confusion.

Also, it is not clear in the abstract that three different groups were considered in the study, which could be addressed as “children under 10 years”, “children with HIV, under 10 years”, and “adults with HIV”.

Response: Thank you for this helpful suggestion. We have revised the **Abstract** to clearly specify the three distinct target groups considered in the study: **“children under 10 years,” “children living with HIV,” and “adults living with HIV.”** This change improves clarity regarding the populations evaluated in our analysis.

It is not clear why the PLHIV target group was not included in the statement regarding the gap in evidence, although it was indicated that existing studies on PLHIV did not include the concurrent testing of LC-aNAATs earlier in that paragraph (lines 132-139, page 5).

Response: Thank you for this helpful comment. We have revised the statement to clarify the evidence gap by explicitly noting that it includes **children, PLHIV, and CLHIV**, ensuring consistency with the earlier part of the paragraph. The updated sentence now reads: *“However, there is a gap in evidence regarding concurrent testing of LC-aNAATs in children, PLHIV and CLHIV.”*

In the “Study Design” section, it would be helpful to briefly mention why Malawi and the Philippines were selected as the settings for this study, and also include the information of HIV prevalence among each target groups for both countries.

Response: Thank you for this suggestion. We have revised the **“Study Design”** section to briefly explain the rationale for selecting **Malawi and the Philippines** as representative settings—Malawi as a high TB and HIV burden country, and the Philippines as a high TB burden country with low HIV prevalence. We have also added information on the **HIV prevalence among the target groups** in both countries to provide relevant epidemiological context.

It would be beneficial for the audience to specify what was meant by PLHIV (Does that include only adults or does that also include adolescents?), also to explicitly state why they considered children only under 10 years old.

Response: Thank you for this thoughtful comment. We have clarified in the **Methods** section that **PLHIV** in this study refers specifically to **adults aged 15 years and older**, consistent with standard reporting definitions. Additionally, we have specified that **children under 10 years** were considered in accordance with the **WHO definition of children** for TB diagnosis and treatment guidelines.

The statement in the “Intervention, and Comparator” section was repeated in the first paragraph of “Modeling” section by using different wording.

Response: Thank you for pointing this out. We have revised the text in the **“Modeling”** section to avoid repetition and ensure consistency with the **“Intervention and Comparator”** section. The description of the diagnostic strategies is now streamlined to focus on model structure without restating intervention details already provided earlier.

In Figure 1, LC-aNAAT was written as LC-aNNAT. Individual was written as “indivisual”. Also, the abbreviations “RS”, “RR”, “ATT” should be defined in addition to the other defined abbreviations in the footnotes of that Figure.

Response: Thank you for carefully reviewing Figure 1. We have corrected the typographical errors, including changing **“LC-aNNAT” to “LC-aNAAT”** and **“indivisual” to “individual.”** Additionally, we have updated the footnotes to define the abbreviations **“RS” (respiratory sample), “RR” (rifampicin-resistant), and “ATT” (anti-TB treatment)**. These corrections have been made in both **Figure 1** and the corresponding **supplementary figure** to ensure consistency across all visual materials.

In the “Assumptions”, it would be better to state that at least these samples need to be provided by each group (lines 196-198) since it creates some ambiguity. (i.e., the text suggests that those are the only samples collected, which is not true, because these individuals could also provide respiratory samples.)

Response: Thank you for this important clarification. We have revised the text in the **“Assumptions”** section (lines 196–198) to clearly state that while individuals in the intervention arm were ideally assumed to provide **multiple samples**, including respiratory samples, **at least one alternative sample** (stool for children, urine for PLHIV, and both stool and urine for CLHIV) would be provided if a respiratory sample was not available.

The parameters provided in the Table S1 (Supplementary Table 1) should be consistent. For instance, for the same group of individuals considered in this study, there were different definitions: “children under 5” and “under-five presumptive children”, “presumptive TB children” (The last two definitions should be changed to “children with presumptive TB”). Also, it seems that the name of the last target group should be “CLHIV”, not “children”.
Response: Thank you for this helpful observation. We have reviewed **Supplementary Table 1** and revised the terminology to ensure consistency across the table. Specifically, we replaced **“under-five presumptive children”** and **“presumptive TB children”** with **“children with presumptive TB”**, and corrected the label for the last target group from **“children”** to **“CLHIV”** to accurately reflect the study population.

In Table S2 (Supplementary Table 2), it would be better to cite the actual (published) sources for the parameter values rather than only referring to an ongoing (and I think it is unpublished) systematic review. For the cost parameters (Table S3), my understanding is that values from another ongoing systematic review were used in the study. If so, please cite the references that include the cost values used in the Table S3 (instead of stating “SR” only).

Response: Thank you for this important comment. We have revised **Supplementary Table 2** and **Supplementary Table 3** to cite the **actual published sources** for the parameter and cost values wherever possible, rather than referring only to ongoing or unpublished systematic reviews.

In Line 229: Please change the part “The primary outcome being …” to “The primary outcome is …”.

Response: Thank you for pointing this out. We have revised the sentence on line 229 to read: **“The primary outcome is …”**

In Line 238: The sentence that includes “… additional scenario analyses were performed.” is ambiguous since the main scenarios were not even identified in the manuscript yet (or the text before that sentence is missing).

Response: Thank you for this observation. We have removed the term **“additional”** from the sentence to avoid ambiguity, as the main scenarios had not yet been introduced at that point in the text.

Although DALYs were calculated in the study, the parameters table regarding the disability weights were not provided in the Supplementary tables (or in the main text). Please provide those parameters in the Supplement.

Response: Thank you for this important observation. We have now added a **new supplementary table** listing the **disability weights and corresponding uncertainty intervals** used to calculate DALYs in the analysis. This table has been included in the Supplementary Materials.

The average value of DALYs as a result of using the concurrent testing in the Philippines was given as 0.66 in the text, but it was stated as 0.62 in Table 1. The authors should confirm which number is correct and change one of those values accordingly.

Response: Thank you for catching this discrepancy. We have confirmed that the correct value is **0.62**, and we have updated the text accordingly to ensure consistency with **Table 1**.

On page 14, the authors provided the results of ICER per additional TB case diagnosed and ICER per death averted in the manuscript (lines 310-314), while the table that includes these results was given in the Supplement (as Table S4). It would be easier for the reader to follow, if the table was shown in the main text as well.

Thank you for the helpful suggestion. We have now moved **Table S4** into the **main text** as **Table 2** to improve readability and allow easier interpretation of the results related to ICER per additional TB case diagnosed and per death averted.

On page 18, in line 376, the results were provided in Table S4 (but the text says “in supplementary table 2”), and in line 387, the cost value was reported as $319, while that value was given as $320 in Table 3. Also, on page 22, in line 437, the results were provided in Table S4 (but the text says “in supplementary table 2”).

Response: Thank you for pointing out these inconsistencies. We have updated the references to reflect that the results previously presented in **Supplementary Table 4** are now included in the **main manuscript as Table 2**. Additionally, we corrected the cost value from **$319 to $320.**

For Supplementary Figures 3a & 3b, it would be better if the x-axis tick marks, and the x-axis limits (not the ranges) were consistent.

Response: Thank you for the helpful suggestion. We have revised **Supplementary Figures 3a and 3b** to ensure that the **x-axis tick marks and axis limits** are consistent across both figures, improving visual comparability and clarity.

For the scenario analyses where different respiratory sample production rates were considered, it would be good to add a rationale for choosing the rates as 20%, 50%, and 80%, and for choosing the sensitivity diagnosis ranges as 20%, 40%, and 80% (as shown in the Supplementary Figures 7a & 7b).

Response: Thank you for this helpful suggestion. We have now added a rationale in the **Scenario Analysis** section to clarify the choice of values. The respiratory sample production rates of **20%, 50%, and 80%** were selected to reflect a plausible range observed in programmatic settings, particularly among children and individuals such as PLHIV and CLHIV. Similarly, the clinical diagnosis sensitivity values of **20%, 40%, and 80%** were chosen to represent a plausible range of diagnostic performance and to explore best- and worst-case scenarios under real-world conditions. These clarifications have been incorporated into the manuscript.

In the “Results of Scenario Analysis” section, it would be good to add a sentence to explain why there was a spike between the 50% respiratory sample production rate and the 80% respiratory sample production rate among children in both countries. Similarly, in the other scenario, a sentence to explain the same type of change (between 40% clinical diagnosis sensitivity and 80% clinical diagnosis sensitivity) is needed.

Response: Thank you for this insightful comment. We have added explanatory sentences in the **“Results of Scenario Analysis”** section to clarify the observed spikes. Specifically, we explain that the **increase in ICER between 50% and 80% respiratory sample production** is due to improved diagnostic yield in the comparator arm, which narrows the difference in effectiveness between the intervention and comparator. In contrast, the intervention arm benefits less because alternative samples (e.g., stool or urine) were already contributing to diagnosis. Similarly, the **increase in ICER between 40% and 80% clinical diagnosis sensitivity** reflects the improved ability of the comparator to detect TB without testing, reducing the incremental benefit of concurrent testing. These explanations have been added to improve interpretation of the scenario analysis results.

On page 24, where the authors describe the results of having different comparators, it would be beneficial to mention that the ICER refers to the ICER per DALY averted for clarity, since there were different ICER values in the manuscript. Also, it would be useful to add a few sentences to describe the changes in ICER values for different comparators. In other words, the reason for the increase in ICER values among children and CLHIV groups and the reason for the decrease in ICER values among PLHIV group, when a different comparator was used, need to be explained.

Response: Thank you for this valuable suggestion. We have revised the relevant section on **page 24** to explicitly state that the ICER values presented refer to **ICER per DALY averted**, to avoid confusion given the use of multiple ICER metrics in the manuscript.

We have also added a description to explain the observed changes in ICER values when different comparators were used. Specifically, we clarified that the **increase in ICER values among children and CLHIV** is due to the comparator arm (stool-based LC-aNAAT) assuming that all individuals provide stool samples, which increases diagnostic coverage and reduces the incremental benefit of the intervention. In contrast, for **PLHIV**, the ICER decreased when LF-LAM alone was used as the comparator, due to its lower diagnostic accuracy compared to LC-aNAAT, thereby increasing the relative effectiveness of the intervention. These additions provide important context for interpreting the results across different population groups.

The table on page 24 that was provided as “Table 2” should be changed to “Table 4”.

Response: Thank you, and we have made the change.

Please cite the sources you considered for the statements in the paragraph on page 25 (lines 512-523).

Response: Thank you for this helpful comment. We have revised the paragraph on **page 25 (lines 512–523)** to include appropriate citations supporting the statements regarding the potential benefits of concurrent testing strategies, the long-term value of early diagnosis, and the generalizability of our findings.

In the paragraph (lines 525-535, on page 26), the authors stated that their results were consistent with the other studies, although it is not clear that other studies, which were cited in the manuscript, also focused on the cases in similar TB/HIV prevalence settings. Please mention the effects of the concurrent use of different testing strategies, and explain why the results of the other studies, despite evaluating a subset of diagnostic tests, aligned with this study.

Response: Thank you for this helpful comment. We have revised the paragraph on **lines 525–535 (page 26)** to make it clearer that the studies we cited were not done in exactly the same settings as ours, but they still looked at similar testing approaches. We now explain that the reason our findings are consistent with those studies is because **using more than one test or sample type helps find more TB cases and improves health outcomes**. Even though those studies only looked at some of the tests we included, the results still support our conclusion that **concurrent testing can be a valuable strategy in different settings**.

In the paragraph where the authors discussed the results of one-way sensitivity analysis (lines 537-549, on pages 26-27), it would be useful to explain how using a different comparator for children and CLHIV than PLHIV affected the changes in the ICER values for the corresponding target populations.

Response: Thank you for this important observation. We have revised the paragraph to explain how the use of **different comparators** affected the ICER values across target populations. Specifically, we clarified that for **children and CLHIV**, using LC-aNAAT on stool alone as the comparator assumed that all individuals provided stool samples, which reduced the difference in diagnostic yield between the comparator and the intervention—resulting in a **higher ICER**. In contrast, for **PLHIV**, LF-LAM alone was used as the comparator, which has **lower diagnostic accuracy**, making the intervention relatively more effective and leading to a **lower ICER**. This explanation has now been added

Please explain in which ways including the effects of averted transmission in the modeling approach would increase the health benefits and cost-effectiveness at a population level (as stated in lines 555-557, on page 27).

Response: Thank you for this thoughtful comment. We have revised the paragraph to explain that **including the effects of averted transmission** in the model would likely lead to **greater estimated health benefits and improved cost-effectiveness**. This is because diagnosing and treating TB earlier through concurrent testing can reduce onward transmission in the community, thereby preventing additional future cases and deaths. Accounting for these indirect benefits would increase the overall impact of the intervention at the population level and further strengthen its value.

In the “Conclusion” part, the clinical diagnosis sensitivity could be added to the key factor (as stated in lines 567-570, on page 28).

Response: Thank you for the helpful suggestion. We have revised the **Conclusion** section to include **clinical diagnosis sensitivity** as one of the key factors influencing cost-effectiveness, as identified in the scenario and sensitivity analyses.

General comments: The authors need to check the manuscript thoroughly, and make sure that there are no grammatical errors or typos.

Response: Thank you for the suggestion. We have thoroughly reviewed the entire manuscript and supplementary materials to correct any **grammatical errors, typos, and inconsistencies**. We have also ensured consistent use of terminology and formatting throughout.

In the cost-effectiveness acceptability curves (Figures 3a & 3b, Figures 5a & 5b, and Figures 7a & 7b), it is not clear that the WTP threshold values, as stated in the text, actually correspond to more than 50% of simulations for each group and each country. Please indicate that information on the plots.

Response: Thank you for this helpful comment. We have **adjusted the x-axis range** in all cost-effectiveness acceptability curve figures to improve visibility and make it easier to interpret whether the **willingness-to-pay (WTP) thresholds** correspond to **more than 50% of simulations** for each group and country. This enhancement supports clearer comparison with the thresholds discussed in the text.

The Results section could have been reorganized without having sub-sections for each target group, since the current manuscript has repetitive statements for each target group (with the same outputs, but different values for each group of population considered in the study. As an example, lines 267-273, lines 333-339, and lines 397-402 have a similar text on the analysis of the same outputs for different target groups.). I believe it would be easier to follow to see the results based on outputs, which are given as stratified by target group. (For instance, Table 1, Table 2, and Table 3 could be merged.)

Response: Thank you for this helpful suggestion. We have revised the **Results** section to reduce repetition and improve clarity. Specifically, we have **merged the previously separate tables into a single Table 1** that presents the results stratified by target group.

Rather than providing each Figure separately, the authors could create multi-panel figures that each panel shows the same output for one of the countries, stratified by the target group (For instance, Figures 3a and 3b (children) could be shown side-by-side. Figures 5a and 5b (PLHIV) and Figures 7a and 7b (CLHIV) would be the other panels in the same figure).

Response: Thank you for the helpful suggestion. In response, we have revised the figures to improve clarity and reduce redundancy by **combining related figures into multi-panel layouts**. Specifically, we have created **side-by-side panels** for each country within the same figure, stratified by target group.

Kind regards,

Alice Zwerling

Associate Professor

School of Epidemiology & Public Health

Faculty of Medicine

University of Ottawa

600 Peter Morand Cres. (301E),

Ottawa ON Canada K1G 5Z3
613-562-5800 (x8277)

[azwerlin@uottawa.ca](http://med.uottawa.ca/)
